# Dysregulated Gene Expression: A Candidate Mechanism for Anxiety Disorders

**DOI:** 10.20900/jpbs.20250004

**Published:** 2025-06-25

**Authors:** Dimitri Traenkner, Mary Steinmann

**Affiliations:** 1 Department of Neurobiology, School of Medicine, University of Utah, Salt Lake City, UT 84112, USA; 2 Department of Psychiatry, School of Medicine, University of Utah, Salt Lake City, UT 84108, USA

**Keywords:** anxiety disorders, gene regulation, Genome-Wide Association Studies (GWAS), dysregulated gene expression, polygenic traits, heritability, cis-regulatory elements, epigenetics, comorbidity, neurobiological mechanisms

## Abstract

Anxiety disorders are among the most prevalent and debilitating mental illnesses worldwide. While environmental factors such as early-life stress contribute to their etiology, genetics also plays a crucial role, with a family history increasing susceptibility. Unlike Mendelian traits driven by single gene variants, anxiety disorders appear to follow polygenic inheritance in which multiple genetic variants collectively shape risk. Genome-wide association studies (GWAS) have identified numerous loci linked to anxiety, yet individual variants have small effect sizes and leave much of the heritability unexplained. A clue to resolving this conundrum may lie in the fact that most GWAS hits reside in non-coding regions with characteristics of gene-regulatory elements. This observation raises the possibility that altered expression of otherwise normal genes contributes to susceptibility. Gene-regulatory elements control when and where genes are expressed. Disruption of these elements may contribute to anxiety disorders by subtly altering neuronal signaling and stress-response pathways. Unraveling the role of gene regulation in anxiety disorders presents a promising avenue for improved diagnosis and targeted treatments. This review explores recent advances in the field and their potential for understanding the genetic architecture of anxiety disorders.

## INTRODUCTION

According to the Institute for Health Metrics and Evaluation (IHME), an estimated 4.7% of the global population is actively experiencing an anxiety disorder, more than any other mental disorder [1]. A comprehensive survey in the United States revealed that about one in three people experiences disabling anxiety at some point in life, and about 60% of those affected are women. Achieving a comprehensive understanding of anxiety disorders remains highly challenging, not just in terms of scientific understanding but also in terms of management and treatment. These difficulties arise from the fact that anxiety disorders have variable clinical presentations, are heavily influenced by environmental factors, show comorbidity with other disorders and are therefore often difficult to diagnose. The study of gene regulation offers new opportunities to disentangle the complexity of anxiety disorders and holds promise for easier diagnosis and more effective treatment of these conditions. Here we review recent insights, advances, concepts, and ideas.

## NORMAL ANXIETY AND ANXIETY DISORDERS

Anxiety is a natural and adaptive response to stress, characterized by feelings of tension, worry, and physiological changes such as increased heart rate. It plays a crucial role in alerting individuals to potential threats and preparing them for action [2]. However, when anxiety becomes excessive, persistent, disproportionate to the situation, and contributes to maladaptive behaviors that impact daily functioning, it may indicate an anxiety disorder. Anxiety disorders are among the most prevalent and debilitating forms of mental illness. They include generalized anxiety disorder, panic disorder, social anxiety disorder, or specific phobias like excessive fear of height or spiders [3,4]. In the United States, 19% of adults are affected by an anxiety disorder annually, with a higher prevalence in women (23.4%) than in men (14.3%) [4]. Adolescents aged 13 to 18 are even more strongly impacted, with 38% of females and 26% of males experiencing disabling anxiety during this period of their lives. The fact that anxiety is a suicide risk factor further emphasizes the urgent need for early diagnosis and effective treatment [5,6]. The chance of developing an anxiety disorder does not appear to depend on a single dominant factor, but rather a combination of neurobiological, environmental, and genetic factors [7–9]. The neurobiological factors thought to contribute are aberrant tuning, processing, and signal transmission of brain areas normally involved in forming healthy stress responses [10]. However, it is often difficult to resolve if neurobiological factors are cause or consequence of an anxiety disorder. In contrast, environmental factors like early-life stress and trauma, such as abuse or neglect, are powerful environmental risk factors and can precipitate anxiety disorders [11]. Genetics unarguably also contributes to the etiology of anxiety disorders and heritability estimates range between 30% to 50%. In other words, an individual’s risk of developing an anxiety disorder is increased if there is a family history of similar disorders [12]. Genome-wide association studies (GWAS) have identified several dozen genetic loci and over 100 associated genes linked to anxiety disorders as both risk or protective factors [13,14]. However, effect sizes of individual loci and associated gene variants are small and contribute only marginally to the overall risk. Even though individual effect sizes are small, the identified gene variants could highlight potential disease mechanisms. The data do not suggest a single dominant process but rather involvement of a broad spectrum of neuron types and signaling mechanisms in the disease etiology [15]. Distributed roots of anxiety disorders are further supported by studies that directly investigate the link between candidate gene variants and anxiety-related behavioral traits in animal models [13,16–19]. For these reasons, anxiety disorders are considered polygenic traits, meaning that multiple gene variants need to combine for a more serious increase in susceptibility. In summary, the consensus is that anxiety disorders are complex heritable phenotypes [13].

## THE ROLE OF GENE REGULATION IN ANXIETY DISORDERS

**Figure 1.**
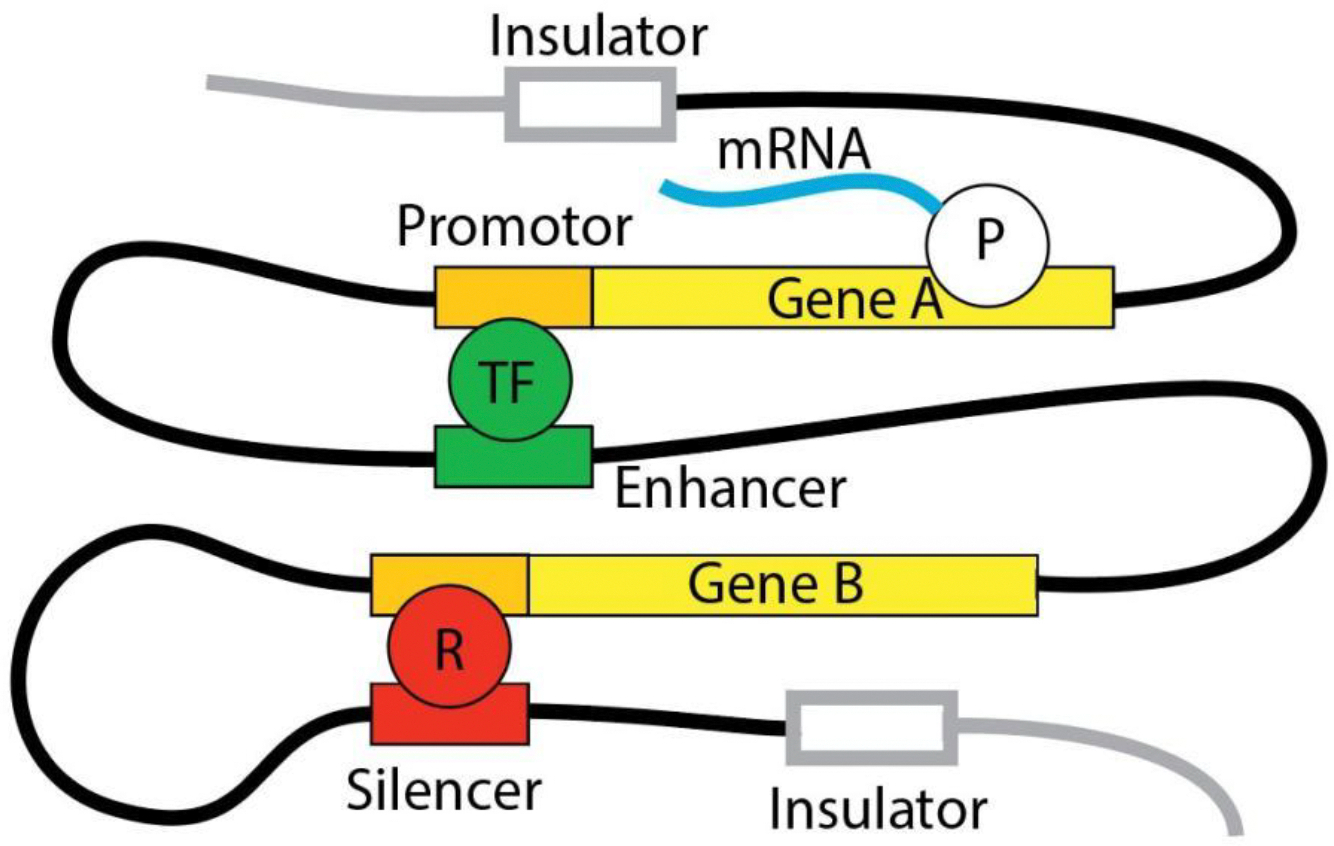
Gene Regulation Overview. Gene regulation is accomplished by various DNA segments outside the gene proper (cis-regulatory elements) and diffusible factors (trans-regulatory elements). Cis-regulatory elements are, based on their effect on gene expression, promotors, enhancers, silencers, or insulators. Promotors typically begin within 5 kilobases (kb) upstream of a gene’s transcription start site and contain binding sites for the core transcription machinery consisting of RNA polymerase and general transcription factors. Promotors also contain binding sites for diffusible repressors that interfere with transcription. Enhancers and silencers can be located many hundred or thousand kb upstream or downstream of the gene they affect but can get into direct proximity of promotors due to the flexibility of chromatin [30]. Enhancers provide additional binding sites for transcription factors, silencers or repressors. Insulators bind molecules that directly or indirectly build a barrier that limits the reach of enhancers and silencers [46]. All cis-regulatory elements can also bind enzymes that can change the affinity of histones for DNA and, thus, remodel chromatin and influence gene accessibility [47]. Hundreds of cis-regulatory elements may influence a single gene [48]. Tens of thousands diffusible trans-acting regulators, including long non-coding (lnc) and micro (mi) RNAs are thought to be encoded in the human genome [49,50]. Cis- and trans-regulatory elements work together to fine-tune a gene’s expression in time, space, and intensity [51]. P: RNA polymerase II; TF: general or specific transcription factor; R: repressor.

What are the odds that sufficient unfavorable gene variants aggregate to make an anxiety disorder likely? Finding a satisfying answer to this question has troubled geneticists ever since the advent of GWAS about 20 years ago and with an increasing appreciation for the fact that most inheritable diseases are not mendelian but polygenic traits [20,21]. Individual GWAS loci of the majority of recognized polygenic traits have small effect sizes that together explain only a modest fraction of the observed heritability [22]. For example, HDL cholesterol, early onset myocardial infarction, or simply a person’s height all have heritability estimates of up to 60%, yet the proportion of heritability explained for these traits by GWAS is at most about 5%. This conundrum has been referred to as “missing heritability”. For some polygenic traits this conundrum has been resolved. The first solution is that a combination of common gene variants with effect sizes too small for GWAS detection can account for most of the observed heritability and can explain trait variance or severity. Height and HDL cholesterol are examples for which this solution applies [23,24]. A second solution is gene variants or *de novo* mutations with large effect size, but that are too rare to impact GWAS results. Polygenic traits for which this second solution applies are autism [25] or schizophrenia [26]. A third solution is that most GWAS hits for polygenic traits lie within non-coding regions of the genome with many characteristics of gene-regulatory elements [27,28]. Typically, single genes are regulated by multiple enhancers, promoters, silencers, and insulators, all of which act in a combinatorial manner to fine-tune the gene’s spatial and temporal expression [29,30] (see Figure 1). Accordingly, gene-regulatory element variants often have relatively small effect sizes and act in a context-dependent manner, influencing gene expression subtly and specific to cell types or conditions [31]. The heritability attributed to these variants may be fragmented across numerous loci, only detectable when considering broader genomic and epigenomic landscapes. Advances in fine-mapping and functional genomics will help to reveal these elusive contributions, further bridging the gap between the frequency of disease-associated gene variants and heritability estimates [32]. Defective gene-regulatory elements as drivers for disease offer a solution to another conundrum. Many polygenic diseases do not surface as an isolated phenotype, but are comorbid with other diseases [33,34]. It is known that gene-regulatory elements rarely control expression of a single gene [35–37]. In fact, the very advantage of gene-regulatory elements is to balance and differentiate gene expression to drive a particular cell function, by promoting the expression of select genes while suppressing that of others [36,38]. Thus, it is easy to imagine how defective gene-regulatory elements can promote multiple disease pathways simultaneously, a phenomenon referred to as pleiotropy, and serve as interfaces between two distinct polygenic traits [39,40]. Expression quantitative trait loci (eQTL) studies are specifically designed to uncover genetic variants that influence gene expression [41–43]. These studies correlate genetic variants and transcript abundance and reveal loci that regulate gene expression levels in cis (variants located near the regulated gene, typically within the same genomic region, often on the same chromosome) or trans (variants that affect genes located at distant loci, often on different chromosomes). The momentum of eQTL studies with focus on the genetic roots of anxiety disorders is currently building, with very promising leads emerging [44,45].

## THE VALUE OF ANIMAL MODELS

Ultimately, emerging anxiety disorder mechanisms need cross-validation and animal models are invaluable in this process [52]. In particular, mouse models offer several advantages. These advantages include that brain circuits underlying stress responses are conserved between mice and humans [53]. These shared circuits, such as the hypothalamic-pituitary-adrenal (HPA) axis or the amygdala-prefrontal pathways, enable translational insights into neural mechanisms driving anxiety. Moreover, diverse behavioral tests that precipitate stress-induced phenotypes in mice that model human anxiety symptoms are well-established, like the elevated plus maze or open field test [54,55]. Most importantly, an expansive toolkit for genetic manipulation in mice facilitates precise dissection of the molecular, cellular, and circuit-level mechanisms underlying anxiety disorders and will set the stage for precision interventions and treatments in humans. Examples for important insights gained from mouse models is that expression levels of brain-derived neurotropic factor (BDNF), a secreted protein linked to anxiety phenotypes, are controlled by epigenetic regulation via promoter methylation [56] or that NPTX2 controls the expression of genes that respond to stress-hormones [57]. Other valuable animal models with established behavioral tests for stress responses are invertebrates like the fruit fly *Drosophila melanogaster* or the nematode *Caenorhabditis elegans*. Although evolutionary farther removed from humans than mice, both fruit fly and nematode offer the benefit of completely mapped connectomes that can be explored with almost limitless options for genetic manipulations [58,59]. In contrast, animal models evolutionarily much closer to humans are non-human primates like Rhesus Monkeys that permit examining complex social and environmental influences on anxiety disorders [60]. A second major advantage is that animal models allow for reverse genetic screens. In classic terms, beginning with anxiety disorders as a phenotype and then isolating the underlying genetic causes like in GWAS are forward genetic screens. In contrast, the reverse approach begins with a known mutation and then details the phenotypic consequences. Such a reverse genetic screen led to the discovery that ablation of Hoxb8, a transcription factor with incompletely characterized molecular function, produces severe over-grooming in mice. This phenotype has been carefully distinguished from scratching, for example triggered by itch, and resembles symptoms of human obsessive-compulsive disorder (OCD; [61,62]). Targeted cell-lineage tracing and ablation by us and others suggest that the loss-of-function phenotype is due to dysfunction in a subset of microglia derived from Hoxb8-expressing progenitor cells (‘Hoxb8 microglia’) [55]. A more detailed phenotypic description of mice with deficient Hoxb8 confirmed that it is one of the most robust models for anxiety-symptoms. Aside from over-grooming, Hoxb8-null mice are characterized by elevated physiological stress responses, cortisol levels, and avoidance of exposed spaces. The pathology begins with sexual maturity and is more severe in females. We were able to demonstrate that the anxiety symptoms scale with levels of the female sex-hormones estrogen and progesterone. Intriguingly, Hoxb8 expression peaks during embryonic development and long before pathology onset. Moreover, gene expression in adult Hoxb8 microglia is almost identical to that of other microglia, indicating that this cell type acts during development [63]. One possible scenario is that Hoxb8-lineage microglia prepare the developing brain circuitry for a balanced response to female sex hormones. They normally suppress anxiety and Hoxb8 enables this specific cell function. As a transcription factor, Hoxb8 binds to gene-regulatory elements for the control of gene expression. We are currently characterizing Hoxb8-regulated genes that we will subsequently match with GWAS and eQTL data from humans. In this context, it is encouraging that we found Hoxb8-binding sites disproportionately represented among human GWAS anxiety-risk genes. Notably, however, the *Hoxb8* gene itself has not yet been associated with anxiety disorders in GWAS. It remains possible that *Hoxb8* mutations are rare in humans or that other disease phenotypes caused by loss of Hoxb8 function obscure anxiety-related symptoms.

Several additional animal models have also helped establish a causal role for gene regulation in anxiety-related behaviors. For example, targeted silencing of the *HTR2A* gene, which encodes the serotonin receptor 5-HT2A, using a noninvasive CRISPR-Cas9 system led to a marked reduction in both *HTR2A* expression and anxiety-like behavior in mice, directly demonstrating a functional role for gene repression in modulating anxiety [64]. Similarly, deletion of a conserved enhancer element (BE5.1) regulating *BDNF* expression resulted in increased anxiety-like behavior, particularly in female mice, underscoring the importance of non-coding regulatory variants in sex-specific susceptibility [65]. In a tauopathy model, CRISPR-based epigenetic activation of *Gad1*, which encodes a GABA-synthesizing enzyme, restored GABAergic synaptic transmission in the prefrontal cortex and significantly improved spatial memory performance [66]. Although this study did not assess anxiety-like behavior directly, the targeted rescue of inhibitory signaling in a brain region strongly implicated in emotional regulation lends mechanistic support to the idea that dysregulated gene expression in GABAergic pathways can influence anxiety. Finally, naturally occurring copy number variation at the *Glo1* locus has been shown to modulate anxiety-like behavior by altering levels of methylglyoxal, a metabolite that acts as a GABA_A_ receptor agonist—thus linking gene dosage and metabolic regulation to neural inhibition and anxiety [67]. This set of examples is not meant to be exhaustive, but together they support the view that dysregulated gene expression—whether through enhancer deletion, transcriptional silencing, epigenetic modulation, or gene dosage imbalance—can act as a causal driver of anxiety-related behaviors in vivo.

## NEW OPPORTUNITIES IN ANXIETY DISORDER RESEARCH

As outlined in the previous paragraphs, aberrant gene regulation has to be considered as a root cause for the non-mendelian inheritability, pleiotropy, and comorbidity of anxiety disorders. Moreover, stress-response circuits spanning multiple brain regions may exhibit exaggerated activity due to a common defect in gene regulation. There are yet other aspects of complex disorders frequently neglected that deserve more attention and may also be addressed in the context of gene regulation. Many internalizing mental disorders, including anxiety and depressive disorders, show similar prevalence in both sexes prior to the onset of puberty but a higher prevalence in females after onset of puberty that persists through the reproductive years [68]. In fact, being female is considered a risk factor for development of anxiety and depression [69,70]. This phenomenon is not understood but has traditionally been explained by the complex interplay of psychological, social, environmental, and biological factors that affect males and females differently [71]. Interestingly, studies examining prevalence of depression and anxiety globally and across cultures show similar gaps in the prevalence of internalizing mental disorders between males and females. Similarly, peak age of onset for anxiety disorders around the time of puberty appear not to be tied to socioeconomic factors, further emphasizing the role of biological sex and hormone changes in developing anxiety or depression [72,73]. Proposed mechanisms of female anxiety independent of hormones include sex chromosome-dependent gene expression and brain development [74]. Hormone-dependent mechanisms of anxiety disorders and other internalizing mental disorders in pubertal and post-pubertal females are currently also mostly speculative. Proposed mechanisms consider the indirect effect of hormonal changes during puberty on body morphology, or the direct effect of estrogens and androgens on brain activity [75–77]. Common to all proposed models trying to explain the sex-bias in anxiety disorders is that females and males are differently affected by a combination of dissociable and independent factors. But what if there is a common root to all these phenomena, inheritability, delayed onset, and sex-bias? Mice with ablated transcription factor Hoxb8 set an example of how this is possible. Expression of Hoxb8 ceases in-utero but anxiety-like symptoms do not appear until onset of sexual maturity [55,63]. Moreover, the pathology in Hoxb8 knock-out mice is more severe in females than in males, is attenuated by treatments that lower female sex-hormones, and can be exaggerated in males by supplementing progesterone and β-estradiol [55]. Together, these findings suggest a mechanistic link between genetic changes and being female in the development of anxiety. One way to interpret these findings is that Hoxb8-dependent cells already completed their protective function in adolescent or adult mice. It is possible that Hoxb8-dependent cellular mechanisms prepare the developing brain circuitry for a balanced response to sex hormones later in life. This could be accomplished, for example, by limiting the sensitivity of neurons for estrogen and progesterone [78–80]. Our current research is exploring these possibilities. On a final note, we want to highlight another curious observation in Hoxb8 knock out mice. Hoxb8 dysfunction produces over-grooming in both genders but marked anxiety-like responses only in females [55]. These observations suggest that OCD- and anxiety-like pathologies are related, but dissociable. This matches the recognized but still unresolved relationship between OCD and anxiety in humans [81] and is yet another example of how the study of gene-regulation can further help to disentangle complex traits. In general, a promising future direction lies in the integration of human GWAS findings with reverse-genetics approaches in mouse models. While GWAS highlights genetic loci associated with anxiety risk, these data often lack functional resolution and thus do not directly instruct how to dissect the underlying disease mechanisms. Conversely, mouse models enable precise manipulation of specific genes or regulatory elements but cannot capture the full spectrum of human anxiety symptoms. By systematically testing the molecular and cellular consequences of GWAS-identified variants in mice—particularly those in non-coding regulatory regions—researchers can validate candidate risk genes, uncover their roles in brain development and stress responses, and model sex-specific or developmental effects. This cross-species strategy can also help identify the regulatory networks and cell types most affected by risk variants, thereby bridging the gap between statistical associations and biological mechanisms. For example, human GWAS data can be intersected with single-cell chromatin accessibility maps (e.g., from scATAC-seq experiments) in mouse brain to pinpoint cell types where anxiety-associated variants are likely active. These candidate regulatory elements can then be functionally tested in vivo using CRISPR interference (CRISPRi) or activation (CRISPRa) in specific brain cell types—such as amygdala-projecting neurons or microglia—to assess their impact on gene expression and anxiety-related behaviors. Ultimately, such integrative approaches may accelerate the translation of genomic findings into mechanistic insights and therapeutic targets for anxiety disorders.

## CONCLUSIONS

Anxiety disorders are among the most prevalent and debilitating mental illnesses, influenced by a complex interplay of genetic, environmental, and neurobiological factors. Recent advances in gene regulation research offer new insights into the etiology, comorbidity, sex-bias, and heritability of anxiety disorders. Emerging evidence suggests that a better understanding of how gene-regulatory mechanisms contribute to anxiety disorders will be a milestone in the search for improved diagnosis and targeted therapeutic interventions.

## Data Availability

No data were generated from the study.

## ABBREVIATIONS

GWAS: Genome-Wide Association Studies
HDL: High-Density Lipoprotein
eQTL: Expression Quantitative Trait Loci
CRISPR: Clustered Regularly Interspaced Short Palindromic Repeats
BDNF: Brain-Derived Neurotrophic Factor
NPTX2: Neuronal Pentraxin 2
OCD: Obsessive-Compulsive Disorder
kb: Kilobase (unit of DNA length)
lncRNA: Long Non-Coding RNA
miRNA: MicroRNA
RNA: Ribonucleic Acid
TF: Transcription Factor
R: Repressor
scATAC: single cell assay for transposase-accessible chromatin using sequencing
